# Evolution of Atomic-Level Interfacial Fracture Mechanics in Magnesium–Zinc Compounds Used for Bioresorbable Vascular Stents

**DOI:** 10.3390/ma17194734

**Published:** 2024-09-26

**Authors:** Zhen Zhou, Chaoyue Ji, Dongyang Hou, Shunyong Jiang, Yuhang Ouyang, Fang Dong, Sheng Liu

**Affiliations:** 1 The Institute of Technological Sciences, Wuhan University, Wuhan 430072, China; zhen.z@whu.edu.cn (Z.Z.); 2018106520021@whu.edu.cn (C.J.); 2022106520011@whu.edu.cn (D.H.); 2023206520002@whu.edu.cn (S.J.); 2022206520021@whu.edu.cn (Y.O.); 2 Wuhan Institute of Quantum Technology, Wuhan 430206, China; 3 School of Power and Mechanical Engineering, Wuhan University, Wuhan 430072, China

**Keywords:** bioresorbable vascular stents, intermetallic compounds, magnesium–zinc interface, molecular dynamics

## Abstract

Bioresorbable magnesium-metal vascular stents are gaining popularity due to their biodegradable nature and good biological and mechanical properties. They are also suitable candidate materials for biodegradable stents. Due to the rapid degradation rate of Mg metal vascular scaffolds, a Mg/Zn bilayer composite was formed by a number of means, such as magnetron sputtering and physical vapor deposition, thus delaying the degradation time of the Mg metal vascular scaffolds while providing good radial support for the stenotic vessels. However, the interlaminar compounds at the metal interface have an essential impact on the mechanical properties of the bi-material interface, especially the cracking and delamination of the Mg matrix Zn coating vascular stent in the radially expanded process layer. Intermetallic compounds (IMCs) are commonly found in dual-layer composites, such as Mg/Zn composites and multi-layer structures. They are frequently overlooked in simulations aiming to predict mechanical properties. This paper analyses the interfacial failure processes and evolutionary mechanisms of interfacial fracture mechanics of a Mg/Zn interface with an intermetallic compound layer between coated Zn and Mg matrix metallic vascular stents. The simulation results show that the fracture mode in the Mg/Zn interface with an intermetallic compound involves typical ductile fracture under static tensile conditions. The dislocation line defects mainly occur on the side of the Mg, which induces the Mg/Zn interfacial crack to expand along the interface into the pure Mg. The stress intensity factor and the critical strain energy release rate decrease as the intermetallic compound layer’s thickness gradually increases, indicating that the intensity of stress and the force of the crack extending and expanding along the crack tip are weakened. The presence of intermetallic compounds at the interface can significantly strengthen the mechanical properties of the material interface and alleviate the crack propagation between the interfaces.

## 1. Introduction

Coronary disease represents the leading cause of premature death worldwide and a leading cause of healthcare expenditures [1]. On a global scale, ischemic heart disease kills over 6 million individuals every year. The World Health Organization predicts this condition to become the most significant single-disease cause of death worldwide, by an increasing margin, in the 2030s [1]. Coronary atherosclerotic heart disease (CAHD) has a high incidence and mortality rate and poses a significant threat to human life and health [2]. Coronary stent implantation has high safety and low surgical trauma, can promote the postoperative recovery of patients with CAHD, and can achieve sound therapeutic effects. As a result, it plays a vital role in the clinical management of CAHD [2]. Magnesium (Mg), a biodegradable material, has been used to construct a promising new generation of vascular stents [3]. The critical advantage of Mg-based materials over others, in terms of coronary stent materials, is their potential to significantly reduce or even eliminate late restenosis, which frequently occurs in permanent stent materials [3]. These stents have demonstrated excellent biocompatibility, and clinical trials have shown promising results [4].

However, the critical challenge associated with Mg for stent applications is its relatively rapid biodegradation, which occurs as a result of corrosion [4]. Rapid corrosion can lead to a loss of mechanical integrity and the release of high concentrations of degradation products [5]. Therefore, most experts focus on studying anticorrosive coatings for biodegradable Mg-based vascular stents, such as polytrimethylene carbonate. This material has recently been applied as a drug-eluting coating on Mg-based alloys, where it has shown surface erosion properties, the ability to mediate stable drug release, and good biocompatibility, thus representing a promising candidate as a drug-eluting coating for magnesium-based stents [6]. The slowing of the corrosion, hemocompatibility, and cytocompatibility of the arginine-leucine-based poly (ester urea urethane) coating indicate that the newly developed family of pseudo-protein biodegradable copolymers may have the potential to offer greater protection and functionality for Mg alloy-based implantable materials, and may bring the use of fully biodegradable Mg-based cardiovascular stents closer to becoming a clinical reality [7]. The design of a zinc (Zn) barrier layer makes it possible for the IBS scaffold to have both ultra-thin struts and a thick poly(D-lactic acid) coating, allowing it to maintain adequate scaffolding after three months of implantation, as well as a significantly shortened corrosion period of 13 months [8].

However, most research on the subject thus far has focused on the corrosive properties of Mg alloy vascular stents, with far less focus on the stent–coating interface’s mechanical properties. Delamination and fractures can occur at this interface when such stents expand to act as scaffolds and keep blood vessels open. The process of polymer coating delamination and stress concentration can be monitored via scanning electron microscopy [9].

Therefore, studying the interface mechanics between the coating and Mg-based vascular stents may have an essential impact on the mechanical properties of the coating process. However, the Zn-Mg interfacial compound layer is generated between the Zn coating and the Mg metal. The morphology of Zn in the coating changes from bulk to strip and finally to mesh-like, while the MgZn_2_ layer changes from rod-like to mesh-like as the Mg content increases [10]. Several methods, such as magnetron sputtering and physical vapor deposition, can obtain a composite coating with IMCs containing Mg-based Zn-coated vascular stents. The composite coating has been obtained and observed in practical applications by relevant means [10,11,12,13].

This study used MD simulation to analyze the intermetallic compound (IMC) layer’s effect on the properties of a dual-layer Mg/Zn composite. We used the MD simulation to set up the Mg/Zn interface model with IMC. Its stress–strain curve, stress contour, and atomic details were used to analyze its crack propagation mode under biaxial tension. Meanwhile, its critical energy release rates, stress intensity factor, and fracture energy were used to investigate the effect of different IMC layer thicknesses on the mechanical properties of the Mg/Zn interface.

## 2. Modeling and Simulation Methods

### 2.1. Force Potential

To study the mechanical properties of the Zn/Mg interfacial layer through molecular dynamic simulation at the atomic scale, values for various functional parameters of the interaction between the Mg-Zn atoms were assigned based on previous publications in the literature [14] that explored the modified embedded-atom method of interatomic potential. The ideal values for the cohesive energy, equilibrium nearest-neighbor distance, and bulk modulus of the HCP Zn structure were calculated via density functional theory, using the Vienna ab initio simulation program (VASP) [15,16,17] with the projector augmented wave (PAW) method [18] and the Perdew–Burke–Ernzerh approach to generalized-gradient approximation (GGA) [19]. The binary potential parameters of different Zn/Mg compounds were optimized by fitting fundamental material properties from experiments and other calculations for relevant systems [14]. These have been successfully applied in other studies [20,21]. Therefore, this work chooses the optimized binary embedded-atom potential [14] to depict the interatomic potential of the magnesium–zinc compounds.

### 2.2. Simulation Model

**Figure 1:**
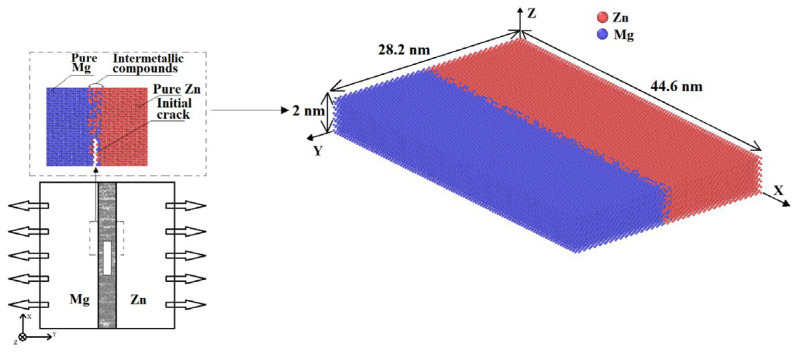
An extrusion model of the Mg/Zn interface with the IMCs.

In this study, the dimension of the simulation model was 44.6 × 28.2 × 2.0 nm^3^ for the unit cell system [22]. The thickness of the IMC layer was set to 1 nm for the MD simulation. An initial crack tip with a length of 3 nm was positioned at the IMC interface’s center, facilitating perfect cleavage along the Mg/Zn interface. The stretching method we use is static stretching along the *Y*-axis. The Mg/Zn interface with IMC was programmed as shown in Figure 1.

### 2.3. Simulation Method

The relaxation temperature of the binary Mg-Zn system was 300 K, and the isothermal–isobaric (NPT) ensemble was then used to increase the temperature to 300 K in zero-pressure conditions [23]. The energy minimization was performed using the conjugate gradient algorithm [24]. To better characterize the mechanical parameters of the material, we used quasi-static stretching at a strain rate of 5 × 10^−8^/s. The direction of biaxial stretching was in the *y*-axis direction. Molecular dynamics simulations were carried out using LAMMPS stable version 2022 software [25], and the results were visualized using Ovito 3.10.6 visualization software [26].

## 3. Results and Discussion

### 3.1. Stress–Strain Response

**Figure 2:**
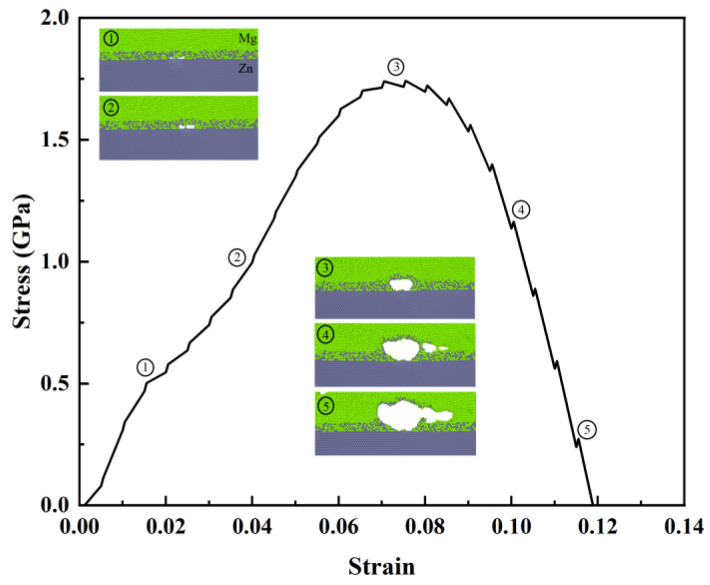
Tensile stress–strain curves at different stages of Mg/Zn interface with IMCs.

The thickness of the IMC layer was set to 1 nm for the MD simulation. The engineering strain and virial stress [27] were calculated for the stress–strain curves of the Mg/Zn interface and its representative atomic configuration throughout the tensile process, as is shown in Figure 2—the elastic phase is labeled as ①, the plastic phase is labeled as ②, the strengthening phase is labeled as ③, and the different degrees of the failure phases are labeled ④ and ⑤. The elastic phase ① is short, and the plastic phase ③ is long. A large plastic plateau can be seen in the stress–strain curve of the figure. Moreover, stress slowly decreases to 0 MPa after the ultimate tensile strength point, and the fracture mode of the Mg/Zn interface with IMCs shows a very pronounced ductility. From Figure 2 ①–⑤, it can be seen that the whole crack is located in the center of the IMCs and gradually becomes biased toward the Mg side. By contrast, the atoms of the Zn layer are only irregularly wavy at the interface. As the strain increases, the crack gradually expands into the Mg layer. It extends along the interface to form a large fracture zone in the Mg layer before finally completing a total separation of the interface. Based on the overall tensile results, the presence of the IMC layer prevents the interfacial fracture propagation from happening at the interface during the stretching process. Instead, the crack propagation occurs from the interface towards the inside of the Mg bulk end.

**Figure 3:**
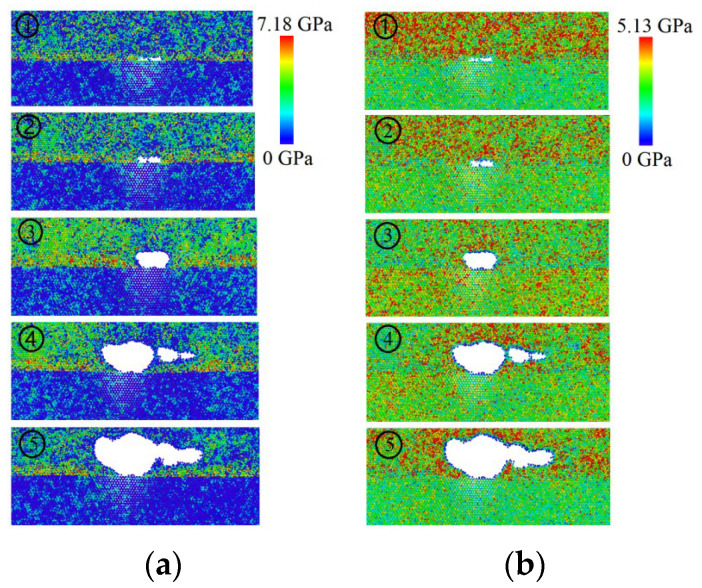
Stress contour diagram at different stages for Mg/Zn interface with IMCs: (**a**) principal stress along the y direction and (**b**) von Mises stress.

Mises stress emphasizes the effective representation of metal fracture failure modes and applies it to nano-micro scale stresses [28,29]. We extracted the Mises stress for each atom from Equation (1). Figure 3a,b show the distribution clouds for the Mises and normal stress (the principal stress in the y direction) for the five phases (i.e., the points labeled ①–⑤ in Figure 2).

(1) $$\sigma =\sqrt{3(\sigma_{xz}^{2}+\sigma_{xy}^{2}+\sigma_{yz}^{2})+(1/2)[{\left(\sigma_{xx}-\sigma_{yy}\right)}^{2}+{\left(\sigma_{xx}-\sigma_{zz}\right)}^{2}+{\left(\sigma_{yy}-\sigma_{zz}\right)}^{2}]}$$

In Equation (1), r_ij_ indicates the component of the virial stress tensor, with the subscripts i and j representing the Cartesian components. Mises and normal stress were calculated for each atom using a LAMMPS code. Visualizations of Mises and normal stress cloud charts in their various phases were generated using OVITO.

Figure 3 shows different stress–strain phases and both normal and von Mises stress. In Figure 3a,b, the Mg layer has a non-uniform structure, whereas the Zn one appears more uniform. From crack nucleation to interface debonding, the Mises and normal stresses are consistently concentrated within the Mg layer. The nucleation occurs at the crack tip and expands into the Mg layer. The von Mises stress concentrates at the Mg layer before the crack nucleation occurs. When the crack tip extends, the von Mises stress concentrates at the crack’s tip in the Mg layer. Notably, the peak stress in the system resulted from breaking the atomic bond. The new crack surfaces came into being while the atomic bonds broke, and the atoms were transferring at the crack under the tensile stress. The crack propagated horizontally in the Mg layer under the tensile stress. When the stress exceeded the critical stress level, the Mg/Zn interface began to debond. The extended and expanded crack then released the stress concentration. Because the tensile strength of the pure Mg layer is lower than that of the pure Zn one, the crack surface propagated along the interface into the Mg layer. In Figure 3a, at the beginning of the crack tip, the principal stress is concentrated around the crack tip contour. With the gradual expansion of the crack and the increase in size, the color around the crack contour gradually becomes blue from the stress cloud diagram, and the principal stress around the crack contour is gradually released so that the stress magnitude gradually decreases. In contrast to Figure 3b, the color around the crack contour line gradually becomes red as the crack propagates, and the Mises stress is concentrated around the crack contour line. The concentration of Mises stress promotes crack propagation, and the principal stresses reflect the stress conditions in the tensile direction.

**Figure 4:**
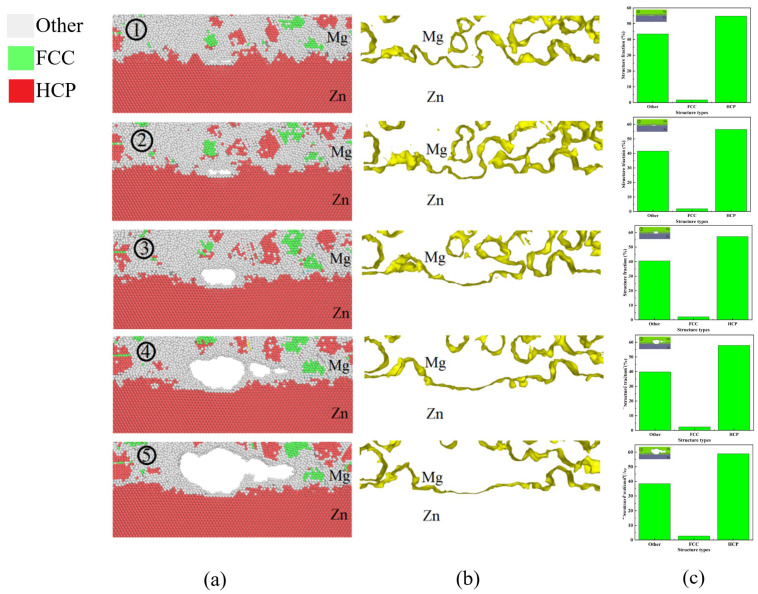
Crystal structure and dislocation diagrams of different stretching stages of the Mg/Zn interface with IMCs: (**a**) The crystal structure of the Mg/Zn interface with IMCs. (**b**) The dislocation line defects of the Mg/Zn interface with IMCs. (**c**) The crystal structure fraction of the Mg/Zn interface with IMCs.

Five stress–strain phases of crystal structure defects are compared in Figure 4, with dislocation defects consistently present in the Mg layer, which correspond to the atomic details in Figure 4b. In Figure 4a, the bonds between the Mg and Zn atoms in the IMC interface gradually weaken from the tensile stress. Meanwhile, wavy streaks form at the interface because of lattice mismatch. As more tensile load is applied, the stress becomes concentrated at the crack tip before moving along the tail of the crack in the interface. The dislocation at the Mg layer exacerbates the dislocation slip between the grain boundaries, leading to crystal defects. These defects in the Mg layer then expand and converge with the interfacial cracks, causing the cracks to extend toward the Mg layer and eventually cause interfacial cracking. Concurrently, crack nuclei are generated inside the Mg layer and gradually expand, eventually forming a crack zone with the cracks at the interface perpendicular to the tensile direction. In Figure 4c, although crystal defects occur during the stretching process, the crystal structure type does not obviously change from the crystal structure content perspective.

**Figure 5:**
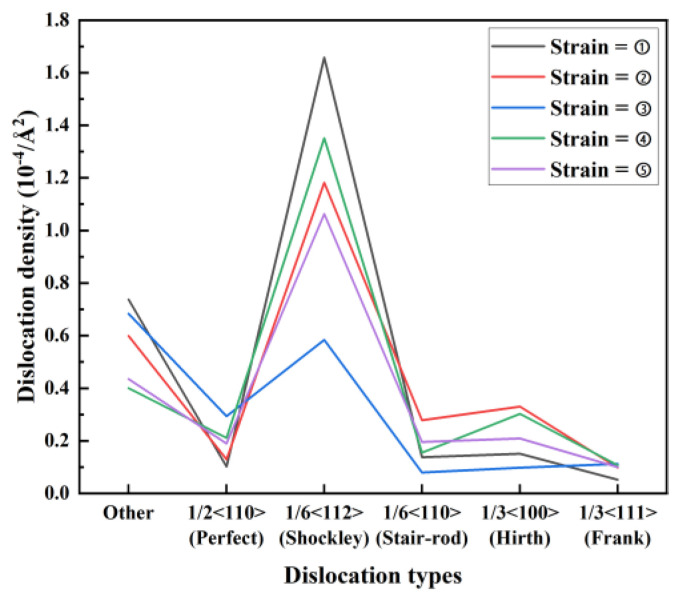
Dislocation density for different dislocation types curve under different strains.

Figure 5 shows the dislocation density distribution for different strains, corresponding to the atomic details presented in Figure 2 (points ①–⑤). The Burgers vector 1/6 < 112 > (Shockley) dislocation density is the largest under different strains except for when the strain = ③, which corresponds to the atomic details in Figure 2 (points ①–⑤). In Figure 2③, when the Mises stress reaches the ultimate tensile strength, the interface is in the stage of imminent fracture, and the Burgers vector 1/6 < 112 > (Shockley) dislocation density changes the most. The main change in the Burgers vector 1/6 < 112 > (Shockley) dislocation density showed the main contributor to dislocation slip along the crystal boundary. The Burgers vector 1/6 < 112 > (Shockley) increase in dislocations is an essential indicator of fracture at the adjacent interface of the Mg-Zn interface with IMCs.

### 3.2. Normal Traction-Displacement Response

The average stress on the atoms along the y direction is defined as σ_yy_. A given atom’s displacement length along the y direction at the IMC interfacial center is denoted as Δy. This study extracted the σ_yy_ and Δy values for different atoms at the center of the IMC interface. Stress contours and opening profiles in the y direction of the IMC interface were extracted along the x coordinate for specific instants.

**Figure 6:**
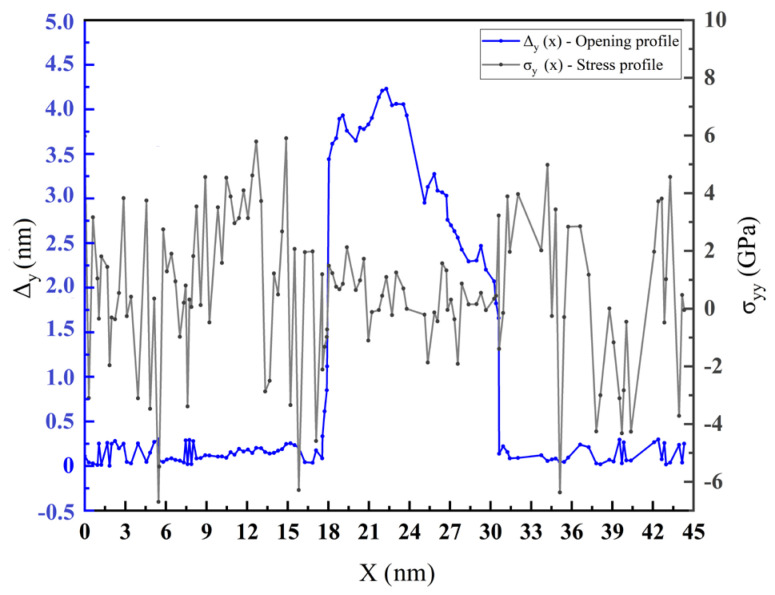
Stress contours in the y direction and opening profiles of the IMC interface, extracted along the x coordinate for specific instants.

Based on the data presented in Figure 6, it is apparent that the crack center located at the interface exhibited the most significant opening profiles. Moreover, the average stress fluctuation along the y direction fell within a relatively flat range between −2 and 2 GPa. The main reason is the release of concentrated stresses by the crack’s expansion and fracture opening in the y direction. The stress concentrated and fluctuated over a wide range on both sides of the crack tip. The crack tip propagation of the Mg-Zn interface with IMCs relieves the stresses around the crack profile. The concentration of stress predicts the dynamic trend of the crack propagation crack.

To better understand how the extension of the crack at the IMC interface responded to traction, we normalized the average stresses (σ_yy_) and the displacement length (Δy) of the atoms at the center of the IMC interface. The σ_yy_/σ_max_ quotient is defined as the non-dimensional effective traction, σ_max_ represents the IMC interfacial critical strength, Δy/Δy_max_ is defined as the non-dimensional effective crack displacement extending along the y direction, and Δy_max_ is the maximum crack displacement along the y direction. The curve of the normalized opening crack displacement along the y direction, as a function of the normalized traction from the simulation model with an IMC thickness of 1 nm, is shown in Figure 6.

**Figure 7:**
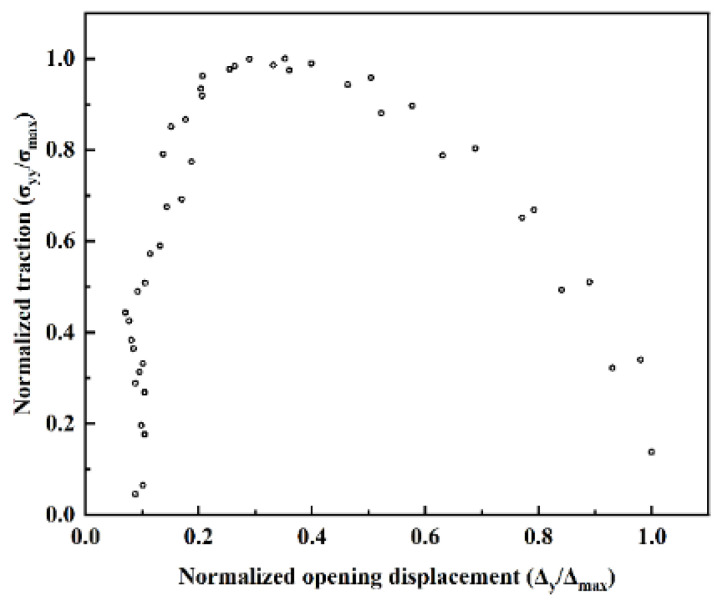
The relationship between the normalized traction and normalized opening displacement.

Figure 7 shows that the normal traction force led to an increase in crack opening. Once the traction force reached critical strength, the interfacial crack extended and expanded to the interfacial failure. As the crack opening displacement increased, the normal traction decreased, and the interface began to fail. As the crack opening displacement increased as a result of interface detachment failure, the deformation within the material released the stresses around the crack. The normal opening displacement was very sensitive to the traction force before the maximum traction was reached but insensitive to this factor after the maximum traction force had been reached. This is consistent with the results in Figure 3, where the principal stress concentrated at the edge of the crack tip contour decreases as the crack propels. In the early stage of interfacial crack propagation, considerable driving stress is required to promote crack initiation. Therefore, the initial normal stress of the Mg-Zn interface with IMC increases instantaneously at the initial stage of crack propagation, and with the gradual increase in the displacement of the crack normal opening, the position of crack propagation extends from the interface to the pure Mg bulk end. It can be seen that the Mg-Zn interface with IMCs can alleviate the interface cracking caused by the initial tensile stress to a certain extent.

### 3.3. Evaluation of Interfacial Strength at Different Calculated Factors

The calculated factors commonly used to measure the strength of interfaces are the stress intensity factor Kc [30] and the critical strain energy release rate Gc [31]. Kc and Gc are shown below in Equations (2) and (3).

(2) $$G_{c}=\int_{0}^{{∆}_{y\max}}\sigma_{yy}d{∆}_{y}$$

Equations (2), σyy, Δy, and Δymax are defined in Section 3.1. The G_c_ variable drives the extension and expansion of the crack and can be interpreted as the energy released per unit area of the crack.

(3) $$K_{c}=\sqrt{G_{c}E}$$

In Equation (2), E is the equivalent modulus, which can be obtained by fitting the stress–strain curves (labeled as ① in Figure 2). The fitting result of the equivalent modulus is shown in Figure 7.

**Figure 8:**
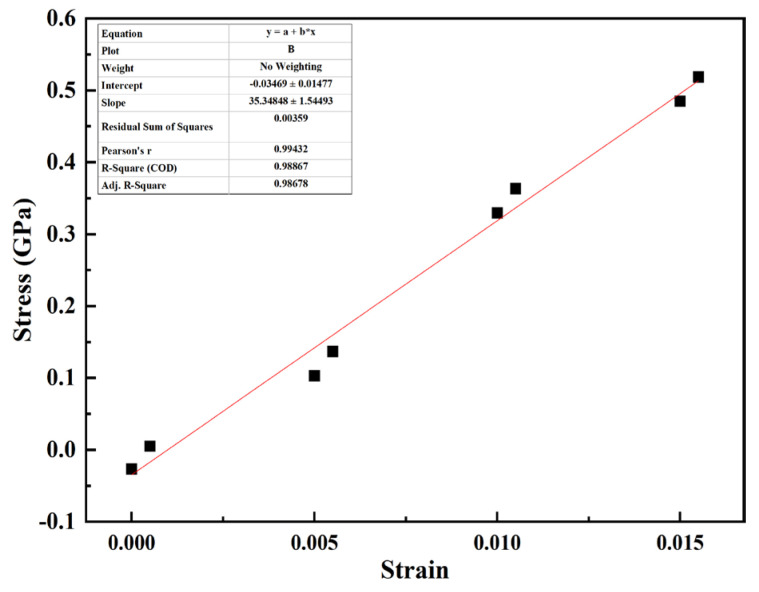
Fitting results of equivalent modulus with IMC thickness of 1 nm.

In Figure 8, the fitting result of the equivalent modulus is 35.35 ± 1.54 GPa, which is near pure Mg and pure Zn (35.6 GPa and 37.1 GPa, respectively [32]). The critical normal stress required for crack propagation is 1.742 GPa, which is near the value for the CNTs/Mg matrix (1 GPa) [33]. Compared to the critical normal stress of the CNTs/Mg matrix required for crack propagation, the mechanical performance of the Mg/Zn interface is better. The work of separation under normal loading equals 0.123 J/m^2^, evaluated by the area under the stress–strain curve. The stress intensity factor K_c_ was calculated at 2.040–2.140 $\mathrm{GPa}\cdot \sqrt{m}$.

### 3.4. Interfacial Mechanical Properties of IMC Layers of Different Thicknesses

**Figure 9:**
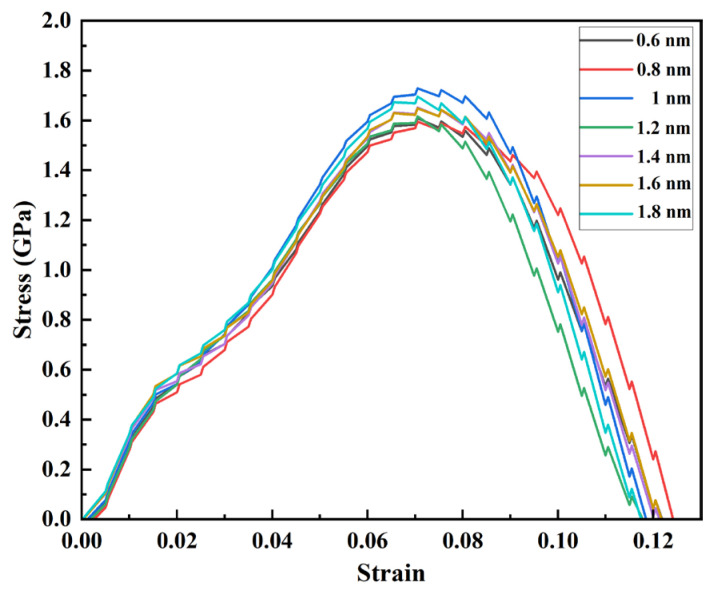
Stress–strain tensile curves for Mg/Zn interface with IMC layer of different thicknesses.

IMC layer thicknesses of 0.6, 0.8, 1, 1.2, 1.4, 16, and 1.8 nm were simulated, and their corresponding stress–strain curves for the Mg/Zn interface are shown in Figure 9.

For IMC layers ranging in thickness between 0.6 and 1.8 nm, there is no noticeable change in the trends already observed throughout different phases of the stress–strain curve. The different stress–strain curves all show a typical plastic fracture mode. The Mg/Zn—IMC interface consistently exhibits a typical ductile fracture pattern with a slow transition from the strengthening phase to the fracture phase. As can be seen from the strengthening phases of the various stress–strain curves, the tensile strengths of most of the thicker interfacial IMC layers are more significant than the thickness of the 0.6–0.8 nm layers. A growing trend is observed regarding tensile strength as the IMC layer’s thickness gradually increases. With the increase in the IMC thickness at the interface, the maximum tensile stress shows an increasing trend, which means that the interlaminar compounds at the Mg-Zn interface can strengthen the mechanical properties between the Mg-Zn interface, which brings the advantage that the risk of delamination between the Zn coating and the Mg matrix is prevented due to the presence of Mg-Zn interface compounds in the dilation process of the Mg matrix Zn coating vascular stent.

In Table 1, the thicknesses of the IMC layer range between 0.6 and 1.8 nm, and the equivalent modulus E is between that of pure Mg and pure Zn (35.83 and 37.1 GPa, respectively) [32]. The change trend remains the same for G_c_ and K_c_. The overall trend is that of G_c_ and K_c_ decreasing as the thickness of the IMC layer increases, indicating that the degree of the concentration of stress along the crack tip is weakened. Thus, it is found that the IMC of the Mg/Zn interface can effectively improve its toughness against fracturing. Increasing IMC thickness is likely beneficial to prevent delamination at the Mg-Zn interface. This indicates that in applying Mg matrix Zn coating vascular stents, the thickness of intermetallic layer compounds of the Mg matrix Zn intermetallic layer can be improved by a number of means, such as magnetron sputtering and physical vapor deposition. This makes the vascular stent-containing Mg matrix Zn coating not only because the Zn coating can effectively resist corrosion but, more importantly because it contains a certain thickness of intermetallic layer compounds, which prevents the risk of interface cracking and delamination between the Mg matrix Zn coating vascular stents during the radial expansion process.

## 4. Conclusions

The fracture mode for a Mg/Zn–IMC interface is a typical ductile fracture. The 1/6 < 112 > Shockley dislocation in the pure Mg contributes to the vast majority of the sliding between the metal lattices during interfacial fracture. The dislocation line defects primarily occur in the pure Mg portion, which induces the Mg/Zn interfacial crack to expand along the interface into the Mg. IMCs can effectively release the stress concentration of the interfacial crack tip by altering the material’s opening and stress profiles. Our calculated result for the equivalent modulus was about 36 GPa, which lies between those of pure Mg and pure Zn (35.6 and 37.1 GPa, respectively) for varying thicknesses of the IMC layer—indicating that the results are reliable. The overall trend in the Gc and Kc parameters is to decrease as the thickness of the IMC layer increases, demonstrating that the intensity of the stress and the force of the crack extending and expanding along the crack tip are proportionally weakened. The IMC interface of a Mg/Zn layer can effectively improve its toughness against fracturing. As the IMC layer’s thickness increases, the fracture’s position gradually extends to the matrix Mg and concentrates on the matrix Mg. The fracture parameters are mainly manifested in the properties of the matrix Mg. The existence of IMCs will be conducive to increasing the fracture toughness of the Mg matrix, but the coating thickness should not be too thick due to the scale effect and the solubility of the over-impedance Mg. Under the action of normal tensile stress, the fracture mechanism of Mg-Zn with IMCs first delays the initiation and propagation of interfacial cracks due to the presence of IMCs, and the cracks first originate at the interfacial IMCs and gradually extend and propagate to the side of Mg until finally the fracture stratification is carried out at the side of the Mg. The interfacial crack is very sensitive to normal stress at the initial stage of IMC propagation at the interface because the Mg-Zn interface contains intermetallic layer compounds, and significant stress is required to promote crack initiation and propagation at the Mg-Zn intermetallic layer compounds. The increase in 1/6 < 112 > Shockley dislocation is an essential marker of fracture strain in the interfacial cracking and layering stage. The dislocation change mainly occurs at the interface and the side of the Mg. The increase in the thickness of intermetallic layer compounds of the Mg matrix Zn intermetallic layer can reduce the concentration of stress intensity at the interfacial crack and delay the initiation and propagation of the interfacial crack, which means that intermetallic layer compounds of Mg matrix Zn intermetallic layers used for bioresorbable vascular stents with a certain thickness can effectively enhance the Mg. The mechanical properties of matrix Zn coating prevent the risk of interface cracking and delamination of Mg matrix Zn coating vascular stents during radial expansion.

## Figures and Tables

**Table 1:** Interfacial fracture parameters for Mg/Zn with varying IMC thicknesses.

T_IMC_	E (GPa)	G_c_ (J/m^2^)	$K_{c}\;(GPa\cdot \sqrt{m})$
0.6 nm	35.45 ± 1.43 [Appendix A]	0.121	(2.029–2.112)
0.8 nm	34.97 ± 1.67 [Appendix A]	0.124	(2.035–2.129)
1.0 nm	35.35 ± 1.54	0.123	(2.040–2.140)
1.2 nm	34.62 ± 1.63 [Appendix A]	0.106	(1.870–2.408)
1.4 nm	35.35 ± 1.54 [Appendix A]	0.115	(1.972–2.060)
1.6 nm	36.54 ± 1.63 [Appendix A]	0.117	(2.021–2.113)
1.8 nm	35.51 ± 1.56 [Appendix A]	0.116	(1.984–2.074)

**Figure A1:**
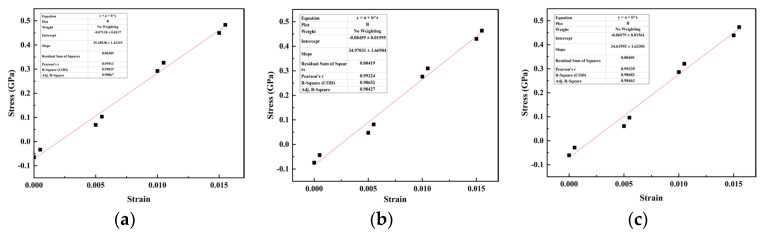

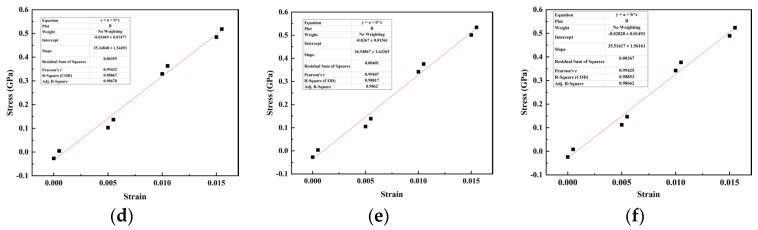
Different fitting results of equivalent modulus G (GPa) with IMC thickness: (**a**) 0.6 nm; (**b**) 0.8 nm; (**c**) 1.2 nm; (**d**) 1.4 nm; (**e**) 1.6 nm; (**f**) 1.8 nm.

## Data Availability

The original contributions presented in the study are included in the article, further inquiries can be directed to the corresponding authors.

## Appendix A

The error bars for the thicknesses of 0.6–1.8 nm are below:

## References

[1] Halim S.A., Newby L.K. (2009). Prognostic biomarkers in individuals with prevalent coronary heart disease. Dis. Markers.

[2] Zhang J., Zhang Q., Zhao K., Bian Y.-J., Liu Y., Xue Y.-T. (2022). Risk factors for in-stent restenosis after coronary stent implantation in patients with coronary artery disease: A retrospective observational study. Medicine.

[3] Zhao N., Watson N., Xu Z., Chen Y., Waterman J., Sankar J., Zhu D. (2014). In Vitro Biocompatibility and Endothelialization of Novel Magnesium-Rare Earth Alloys for Improved Stent Applications. PLoS ONE.

[4] Ma J., Zhao N., Betts L., Zhu D. (2016). Bio-Adaption between Magnesium Alloy Stent and the Blood Vessel: A Review. J. Mater. Sci. Technol..

[5] Staiger M.P., Pietak A.M., Huadmai J., Dias G. (2006). Magnesium and its alloys as orthopedic biomaterials: A review. Biomaterials.

[6] Tang H., Li S., Zhao Y., Liu C., Gu X. (2022). A surface-eroding poly(1,3-trimethylene carbonate) coating for fully biodegradable magnesium-based stent applications: Toward better biofunction, biodegradation and biocompatibility. Bioact. Mater..

[7] Liu J., Wang P., Chu C.-C., Xi T. (2017). Arginine-leucine based poly (ester urea urethane) coating forMg-Zn-Y-Nd alloy in cardiovascular stent applications. Colloids Surf. B Biointerfaces.

[8] Lin W.J., Zhang D.Y., Zhang G., Sun H.T., Qi H.P., Chen L.P., Liu Z.Q., Gao R.L., Zheng W. (2015). Design and characterization of a novel biocorrodible iron-based drug-eluting coronary scaffold. Mater. Des..

[9] Wei W., Petrini L., Altomare L., Farè S., Tremamunno R., Zhentao Y., Migliavacca F. (2014). Modeling and Experimental Studies of Peeling of Polymer Coating for Biodegradable Magnesium Alloy Stents. Rare Met. Mater. Eng..

[10] Dong A., Li B., Lu Y., Zhu G., Xing H., Shu D., Sun B., Wang J. (2017). Effect of Mg on the Microstructure and Corrosion Resistance of the Continuously Hot-Dip Galvanizing Zn-Mg Coating. Materials.

[11] La J., Song M., Kim H., Lee S., Jung W. (2018). Effect of deposition temperature on microstructure, corrosion behavior and adhesion strength of Zn-Mg coatings on mild steel. J. Alloys Compd..

[12] Park J.-H., Ko K.-P., Hagio T., Ichino R., Lee M.-H. (2022). Effect of Zn-Mg interlayer on the corrosion resistance of multilayer Zn-based coating fabricated by physical vapor deposition process. Corros. Sci..

[13] Li L., Wang X., Zhang Z., Qi F., Zhang D., Ouyang X. (2021). Effect of Zn film thickness on corrosion resistance and mechanical properties of WE43 alloy. Mater. Charact..

[14] Jang H.-S., Kim K.-M., Lee B.-J. (2018). Modified embedded-atom method interatomic potentials for pure Zn and Mg-Zn binary system. Calphad.

[15] Kresse G., Hafner J. (1994). Ab initio molecular-dynamics simulation of the liquid-metal–amorphous-semiconductor transition in germanium. Phys. Rev. B.

[16] Kresse G., Furthmüller J. (1996). Efficiency of ab-initio total energy calculations for metals and semiconductors using a plane-wave basis set. Comput. Mater. Sci..

[17] Kresse G., Furthmüller J. (1996). Efficient iterative schemes for ab initio total-energy calculations using a plane-wave basis set. Phys. Rev. B.

[18] Blöchl P.E. (1994). Projector augmented-wave method. Phys. Rev. B.

[19] Perdew J.P., Burke K., Ernzerhof M. (1996). Generalized Gradient Approximation Made Simple Phys. Phys. Rev. Lett..

[20] Jang H.-S., Lee B.-J. (2018). Effects of Zn on <c + a> slip and grain boundary segregation of Mg alloys. Scr. Mater..

[21] Baburao B., Kumar N.H., Edukondalu A., Ravinder D. (2023). Influence of Er/Fe substitution on Mg-Zn nanoparticles’ electromagnetic properties and applications. Braz. J. Phys..

[22] Ji C., Cai X., Zhou Z., Dong F., Liu S., Gao B. (2021). Effects of intermetallic compound layer thickness on the mechanical properties of silicon-copper interface. Mater. Des..

[23] Liu R., Wang J., Wang L., Zeng X., Jin Z. (2022). Cluster Hardening Effects on Twinning in Mg-Zn-Ca Alloys. Metals.

[24] Grippo L., Lucidi S. (1997). A globally convergent version of the Polak-Ribiere conjugate gradient method. Math. Program. Ser. A B.

[25] Plimpton S. (1995). Fast Parallel Algorithms for Short-Range Molecular Dynamics. J. Comput. Phys..

[26] Stukowski A. (2010). Visualization and analysis of atomistic simulation data with OVITO-the Open Visualization Tool. Model. Simul. Mater. Sci. Eng..

[27] Tsai D.H. (1979). Virial theorem and stress calculation in molecular dynamics. J. Chem. Phys..

[28] Ryu S.-K., Lu K.-H., Zhang X., Im J.-H., Ho P.S., Huang R. (2011). Impact of near-surface thermal stresses on interfacial reliability of through-silicon Vias for 3-D interconnects. IEEE Trans. Device Mater. Reliab..

[29] Buehler M.J., Gao H.J. (2004). A mother-daughter-granddaughter mechanism of shear dominated intersonic crack motion along interfaces of dissimilar materials. J. Chin. Inst. Eng..

[30] Irwin G.R. (1957). Analysis of stresses and strains near end of a crack traversing a plate. J. Appl. Mech..

[31] Griffith A.A. (1921). The phenomena of rupture and flow in solids. Philos. Trans. R. Soc. A Math. Phys. Eng. Sci..

[32] Shi H., Xu C., Hu X., Gan W., Wu K., Wang X. (2022). Improving the Young’s modulus of Mg via alloying and compositing—A short review. J. Magnes. Alloys.

[33] Ghasemi M.J., Silani M., Maleki A., Jamshidian M. (2020). Micromechanical simulations and experimental characteristics of randomly distributed carbon nanotubes reinforced Mg matrix composites. Def. Technol..

